# Cross-cultural validation of the Functional Vision Questionnaire for Children and Young People (FVQ_CYP) with visual impairment in the Dutch population: challenges and opportunities

**DOI:** 10.1186/s12874-019-0875-9

**Published:** 2019-12-03

**Authors:** Ellen B. M. Elsman, Valerija Tadić, Carel F. W. Peeters, Ger H. M. B. van Rens, Jugnoo S. Rahi, Ruth M. A. van Nispen

**Affiliations:** 1 Department of Ophthalmology, Amsterdam UMC, Vrije Universiteit Amsterdam, the Amsterdam Public Health research institute, Amsterdam, the Netherlands; 2 School of Human Sciences, University of Greenwich, London, UK; 3 Life Course Epidemiology and Biostatistics Section, Population, Policy and Practice Programme, UCL Great Ormond Street Institute of Child Health, London, UK; 4 Great Ormond Street Hospital for Children NHS Foundation Trust, London, UK; 5 Department of Epidemiology & Biostatistics, Amsterdam UMC, Vrije Universiteit Amsterdam, the Amsterdam Public Health research institute, Amsterdam, the Netherlands; 6 Department of Ophthalmology, Elkerliek Hospital, Helmond, the Netherlands; 7 Ulverscroft Vision Research Group, London, UK; 8 National Institute for Health Research (NIHR) Biomedical Research Centre at Moorfields Eye Hospital NHS Foundation Trust and UCL Institute of Ophthalmology, London, UK

**Keywords:** Visual impairment, Cross-cultural validation, Children, Functional vision, Item response theory, Differential item functioning

## Abstract

**Background:**

To assess cross-cultural validity between Dutch and English versions of the FVQ_CYP, a patient-reported outcome measure developed in the United Kingdom (UK) for children and adolescents with (severe) visual impairment or blindness (VI for brevity) to measure functional vision.

**Methods:**

The 36-item FVQ_CYP was translated and adapted into Dutch using standard guidelines. The questionnaire was administered to Dutch children and adolescents aged 7–17 years (*N* = 253) with impaired vision (no restrictions regarding acuity). Data were compared to existing UK data of children and adolescents aged 10–15 years (*N* = 91) with VI (acuity LogMar worse than 0.48). As with the original UK FVQ_CYP validation, a rating scale model (RSM) was applied to the Dutch data.

**Results:**

Minor adaptations were needed in translation-rounds. Significant differences in item responses were found between the Dutch and UK data. Item response theory assumptions were met, but fit to the RSM was unsatisfactory. Therefore, psychometric properties of the Dutch FVQ_CYP were analysed irrespective of the original model and criteria used. A graded response model led to the removal of 12 items due to missing data, low information, overlapping content and limited relevance to Dutch children. Fit indices for the remaining 24 items were adequate.

**Conclusions:**

Differences in population characteristics, distribution of responses, non-invariance at the model level and small sample sizes challenged the cross-cultural validation process. However, the Dutch adapted FVQ_CYP showed high measurement precision and broad coverage of items measuring children’s functional vision. The underlying reasons for differences between countries in instrument performance are discussed with implications for future studies.

## Background

In recent years, emphasis on patient-centred care has resulted in the development of generic and disease-specific patient-reported outcome measures (PROMs) [1, 2]. Using PROMs, health outcomes such as quality of life, functional status and disease severity, which are preferably reported by patients themselves, can be assessed and monitored [3–5]. Although many vision-specific instruments for adult populations exist (e.g. [6–12]), there is a paucity of such measures in paediatric ophthalmology.

Availability and implementation of instruments to assess functional vision in paediatric ophthalmology would complement objective clinical measures of visual function, such as visual acuity and visual field. Furthermore, these instruments can be used to monitor and evaluate the effectiveness of low vision rehabilitation. Currently, three measures of functional vision are available. Both of the two versions of the LV Prasad-Functional Vision Questionnaire have been developed for children in the developing world [13, 14], and therefore some items have limited applicability in developed countries. The Cardiff Visual Ability Questionnaire for Children and Young People has been developed and tested in a specific geographical area in the United Kingdom (UK), and although translated and validated in Turkish and Chinese [15, 16], its applicability elsewhere is currently limited [17].

The Functional Vision Questionnaire for Children and Young People (FVQ_CYP) for 10 to 15 year olds was previously designed to capture self-reported level of difficulty in the performance of vision dependent activities and is intended for children and adolescents with visual impairment (VI), severe VI or blindness i.e. with acuity in their better seeing eye of logMAR worse than 0.48. It was developed for and validated in a nationally representative sample of UK children [18]. The FVQ_CYP 10–15 years comprises 36 items measured on a 4-point scale. It has good psychometric properties, and is relatively short and easy to complete. Previous analyses, including Rasch analysis, have demonstrated its unidimensionality, reliability and robustness [18]. Extensions of the FVQ_CYP to cover the age range 8 to 18 years are currently in development.

No measure of functional vision is currently available in the Netherlands. Although progress is being made in the development of age-specific versions of the Participation and Activity Inventory for Children and Youth (PAI-CY) [19], there is a need for a reliable and valid measure of functional vision for children and adolescents. Such instrument can be used as a PROM, complimentary to the objective clinical measures of visual function in ophthalmology, as an outcome instrument in research evaluating therapies of interventions, or to monitor and assess the effectiveness of low vision rehabilitation. The aim of this study was to translate the FVQ_CYP in Dutch, including assessment of its cross-cultural validity using Item Response Theory (IRT) analysis. Data for this study was collected as part of a study in which the PAI-CY was developed and its psychometric properties were assessed. The study was performed among a population of children aged 7–17 years with impaired vision from any cause who were registered at a low vision service for a functional vision assessment or various other rehabilitation or early interventions.

## Methods

The cross-cultural validation of the FVQ_CYP was conducted in two phases. The first phase consisted of translation of the FVQ_CYP into Dutch, in keeping with standardized guidelines [20]. The second phase entailed the assessment of psychometric properties of the Dutch version of the FVQ_CYP (FVQ_CYP_NL) using IRT analysis that drew on the existing anonymised UK dataset for the FVQ-CYP.

### Phase 1: translation of the FVQ_CYP into Dutch

The FVQ-CYP was translated in Dutch using an established process for cross-cultural adaptation of PROMs [20]. It comprised five stages outlined below:

#### Initial forward translations

Forward translation from English (source language) into Dutch (target language) was carried out by two independent bilingual translators, having Dutch as native language but were also fluent in English. Both translators were researchers regularly working with visually impaired children and aware of the concept of functional vision, and were as such informed translators. The instructions, questionnaire items, and scale were translated independently without any discussion between the translators.

#### Synthesis of the translations

The two translations were compared and any discrepancies were resolved through discussion and/or consultation of a third researcher not involved in the forward translation. Working from the original FVQ_CYP, as well as from the two translations, a synthesis of these translations was produced, resulting in one common translation.

#### Back translations

The translated version of the FVQ_CYP was then translated back to English by two bilingual translators who were native English speakers. The two back-translators were naïve to the original English version of the FVQ_CYP and lay to the concept of functional vision and VI.

#### Expert committee review

An expert committee including the project leader and all four translators reviewed all translations and resolved discrepancies through discussion resulting in consensus on the final wording to be used for the final version of the Dutch FVQ_CYP (FVQ_CYP_NL).

### Phase 2: assessing psychometric properties of the FVQ_CYP_NL

#### Study design and participants

Children and adolescents aged 7–17 years enrolled for care at two Dutch low vision rehabilitation centres at the time of the study or in the past were invited to participate in the study. Children were required to have adequate knowledge and understanding of the Dutch language to participate in the study. Children with registered extensive (cognitive) impairment were excluded from the sample to be invited to participate by the low vision rehabilitation centres. Children with low vision from any cause were eligible and there was no restriction regarding visual acuity. As such, the inclusion criteria were more liberal with respect to both age and visual acuity than for the original instrument development and validation in the UK, which was intentionally limited to children and adolescents aged 10–15 years old with VI/severe VI or blindness i.e. visual acuity in the better eye of logMAR ≥0.48 [18]. More details of the UK sample have been published elsewhere [18]. All eligible Dutch children and their parents were sent a letter explaining the aim and procedure of the study and appropriate consent forms asking whether they would agree to participate. Parents of children who did not respond were telephoned to provide further information about the study and ask for their reasons for declining participation.

Although the original FVQ_CYP is intended for self-administration, Dutch children and adolescents who participated in the study were visited at their home by a researcher in order to administer the FVQ_CYP_NL using an interview format, providing an extra check on ability to participate. Besides, using an interview format was in line with the mode of administration applied for testing the PAI-CY. Parents provided information on socio-demographic and clinical characteristics of their child, such as age, gender, siblings, cause of low vision, level of VI, and other impairments, using a web-based survey questionnaire. Decimal visual acuity, visual field and ophthalmic diagnoses were retrieved from patient files at the low vision rehabilitation organisations. Missing values in patient files were supplemented by self-reported data of parents (*n* = 8). Visual acuity was converted into logMAR, and put into 5 levels based on the better seeing eye, according to World Health Organisation (WHO) taxonomy of VI [21]. VI0 referred to logMAR ≤0.47, VI1 to logMAR 0.48–0.70, VI2 to logMAR 0.71–1.00, SVI to logMAR 1.01–1.30, and blind to logMAR ≥1.31. Thus VI0 was not a category/population for which the FVQ_CYP was designed. When data on visual field was available, visual field of ≤10 degrees was classified as blind; otherwise only visual acuity was used for classification.

The study protocol was approved by the Medical Ethical Committee of the Amsterdam UMC, location VUmc, the Netherlands. The study adhered to the tenets of the Declaration of Helsinki and its later amendments. Written informed consent was obtained from all Dutch participants, i.e. from parents of all children, and from children and adolescents aged 13 years and older. Secondary analysis of the existing anonymised UK FVQ_CYP dataset did not require ethics approval. The data were drawn from the original development and psychometric study which involved individual consent to participation and was approved by the National Health Service Research Ethics Committee for UCL Institute of Child Health and Great Ormond Street Hospital, United Kingdom, and followed the tenets of the Declaration of Helsinki.

#### Statistical analysis

All statistical analyses related to IRT were conducted in R [22]. The remaining analyses were completed using SPSS version 22 [23].

Participants with > 25% missing responses were removed from the analyses. Sociodemographic and clinical characteristics for the Dutch and UK sample were investigated separately.

Item analysis, comprising descriptive statistics of each of the individual items, were conducted for the Dutch and UK samples. Differences in the distribution of responses over the response categories were investigated using chi-square tests.

Following the cut-off criteria used in the validation of the original FVQ_CYP [18], items in the Dutch sample with > 20% missing data were flagged. Moreover, items with > 60% of the responders endorsing the first or last response category (floor and ceiling effect) were flagged, as were items showing certain response categories to be redundant (i.e. not having an answer in a certain response category). Inter-item correlations were evaluated and item-pairs were flagged when correlations were > 0.7, indicating potential redundancy.

Then, IRT was applied on the Dutch sample. IRT comprises a collection of modelling techniques from modern measurement theory. It provides a powerful context to develop instruments which are more efficient, reliable and valid [24]. The statistical models used in IRT analyses describe the association between a person’s ability (latent trait, e.g. disability, denoted as theta (θ)) and the probability of that person choosing a certain response option of an item in a multi-item scale measuring that trait [25]. Application of IRT models requires three assumptions:

- **Unidimensionality**, which assumes that a single latent trait explains the covariance of items [24]. Unidimensionality was assessed by performing an eigen value decomposition on the matrix of robust (Spearman) correlations between the items. A difference approximation to the second-order derivatives along the eigenvalue curve (scree plot) was calculated. This acceleration-approximation indicates points of abrupt change along the eigenvalue curve and the number of eigenvalues before the point with the most abrupt change (the point with the maximum acceleration value) represents the number of latent dimensions that dominate the information content [26]. Subsequently a principal component analyses (PCA) was performed to proxy if all items load on a single component (where the component is taken as a proxy for the latent trait).
- **Local independence of items**, which requires that item responses are independent given their relationship to the latent trait. Local dependence was assessed by inspection of possible excess covariation (> 0.25) among items in the residual matrix resulting from PCA. Local dependence could occur in items that are similar in content, refer to a similar condition (similar stem) or are presented successively [24]. Item pairs with excess covariation were flagged.
- **Monotonicity**, which states that the probability of endorsing a higher item response category should not decrease with increasing levels of the underlying latent trait [27]. Monotonicity was evaluated using Mokken scale analysis. The graphs were visually inspected, and the Loevinger H coefficient was calculated to assess scalability [28] (see also [29, 30]). A Loevinger H coefficient < 0.30 was considered unsatisfactory.

Because on the original FVQ_CYP the rating scale model (RSM) was used [18], this model was also applied to the Dutch data using the eRm package [31]. However, the goodness-of-fit test was not suggestive for satisfactory model fit of the RSM to the Dutch data. Moreover, item misfit was indicated by multiple tests (i.e. graphical model check, Wald test and chi-square fit statistics). Therefore, it was decided to apply another IRT model to the data of the FVQ_CYP_NL. The graded response model (GRM) was selected for this purpose, as it is one of the most commonly used IRT models to evaluate questionnaires with ordinal response categories. It estimates a discrimination parameter (α) and threshold parameters (β) [32, 33]. The thresholds mark the points on the latent trait where the probability of endorsing the response category of an item is 50%, whereas the discrimination specifies the slope of the item curves; the discrimination describes the ability of an item to differentiate between individuals with different trait levels. Using the ltm package [34], model fit of the GRM was assessed by comparing a full model [24] with a constrained model [24, 35], which is nested within the full model and has equal discrimination parameters across all items (analogous to the Rasch model). A Likelihood Ratio test was performed to assess whether the full model fitted the data better than the constrained model. Overall fit of the selected model was assessed using the mirt package [36], yielding several fit indices: root mean square error of approximation (RMSEA), standardized root mean square residual (SRMR), comparative fit index (CFI), and Tucker-Lewis index (TLI). The CFI and TLI should be around 0.95 or higher, whereas the SRMR should be around 0.08 or lower and the RMSEA around 0.06 or lower [37].

Some items might not fit the GRM model, and therefore individual item fit was assessed using the X^2^ statistic [38, 39]. Significance of this test was adjusted for the number of items to correct for multiple testing. Then item information of an item over the latent trait was examined to assess item functioning. Item information refers to the information content of an item in relation to the total test information, and therefore represents reliability or measurement precision [24]. Items with low information across the disability trait were considered for elimination, but the Item Information Curves (IICs) and Category Response Curves (CRCs) also informed decisions, as did content validity. The IICs show the range of the underlying trait over which an item is most useful to distinguish between participants. The CRCs show the relation between the latent trait and the probability of responding to a categorical item [40]. When the curves of two or more items cover the same area on the disability trait, the item with least information and/or holding information over the smallest range of the disability trait was considered for elimination. A person-item map was computed with the WrightMap package to evaluate whether item difficulty matches participants’ ability [41]. It shows the distribution of person parameters (thetas of respondents) on the left side of the map and the distribution of item parameters (thresholds) on the right side.

Differential item functioning (DIF) analyses were used to assess whether participants from different groups (i.e. age and gender) with the same level of disability have different probabilities of selecting a certain response to an item [40, 42]. Two certain types of DIF can be distinguished. Uniform DIF means that an item is endorsed either more or less often at all values of the latent trait by one group compared to the other. Non-uniform DIF does not occur equally at all points on the latent trait, i.e. an item is endorsed either more or less often at some values and the other way around at other values by one group compared to the other [42]. DIF was assessed with the lordif package [43], using an iterative hybrid of ordinal logistic regression and IRT. The likelihood ratio χ^2^ test at α level 0.01 was used as detection criterion, and McFadden’s pseudo R^2^ was used as a measure of magnitude of DIF; a change of 2% was considered as critical value [44]. DIF was evaluated for age (median split: younger than 11 years vs. 11 years and older) and gender (male vs. female).

Known-group validity was assessed to ensure the FVQ_CYP_NL is able to discriminate between groups [42]. Therefore, differences in thetas between groups that differed in level of VI, other impairments and gender were assessed using independent samples t-tests and ANOVA with post hoc Tukey test. VI0 formed the group mild VI, VI1 and VI2 were combined in the group moderate VI, and SVI and blind were combined in the group severe VI/blindness. Participants with unknown levels of vision impairment were excluded from this analysis. It was expected that thetas would increase for increasing levels of VI (signalling worse functional vision), females would have similar thetas as males, and those with other impairments would have higher thetas than those without other impairments.

## Results

### Translation of the FVQ_CYP into Dutch

Some minor differences in wording of two items related to activities at school, i.e. “taking part in science classes” and “seeing the board in the class” were found and were resolved by discussion: examples (i.e. physics and biology) were added to science classes, and board was translated to schoolboard or digital board, as most schools in the Netherlands nowadays use a digital board. During the first questionnaire administration to participants, it was noted that the response option ‘not applicable’ was warranted, because in the Netherlands young children usually do not have homework for which they need the computer, and not all classes (e.g. science and geography) are obligatory for all ages to which the questionnaire was administered. It is worth noting that the ‘not applicable’ option was also included in the original FVQ_CYP UK study, but was subsequently removed due to high endorsement of this category, resulting in a high proportion of ‘missing’ data. Furthermore, it was noted that different wording might be necessary for younger children vs. the older children. For example, math classes were translated into the Dutch word “wiskunde” (i.e. mathematics), but only children in high school have “wiskunde”. In primary school, this class is called “rekenen” (i.e. to calculate). Therefore, two age-appropriate versions (7–12 years and 13–17 years respectively) of the FVQ_CYP_NL were created with minor differences in the wording of five items related to activities at school.

### Participant characteristics

A total of 263 Dutch children and adolescents were recruited in this study. Ten participants were excluded from the analyses because they had an excessive number of missing responses. In the UK dataset, this was the case for three participants, resulting in a dataset containing responses of 91 children and adolescents. The demographic and clinical characteristics of the Dutch sample and the UK sample are summarised in Table 1. As expected there were differences in age, level of VI and occurrence of other impairments, due to more liberal inclusion criteria with respect to these variables in the Dutch sample.

**Table 1:** Socio-demographic and clinical characteristics of the Dutch and the UK sample

	Dutch sample *N* = 253	UK sample *N* = 91
Age in years, mean ± SD (range)	11.06 ± 2.87 (7–18)	12.09 ± 1.84 (9–15)
Gender, n (%)		
Male	150 (59.3)	52 (57.1)
Female	103 (40.7)	39 (42.9)
Level of VI		
VI0: logMAR ≤0.47	126 (49.8)	–
VI1: logMAR 0.48–0.70	56 (22.1)	42 (46.2)
VI2: logMAR 0.71–1.00	35 (13.8)	31 (34.1)
SVI: logMAR 1.01–1.30	4 (1.6)	10 (11.0)
Blind: logMAR ≥1.31	24 (9.5)	8 (8.8)
Unknown	8 (3.2)	–
Nationality		
Ethnic majority	228 (90.1)	77 (84.6)
Ethnic minortiy	25 (9.9)	14 (15.4)
Other impairment, n (%)		
Yes	117 (46.2)	28 (30.8)
No	124 (49.0)	62 (68.1)
Unknown	12 (4.7)	1 (1.1)
Siblings, n (%)		
No	29 (11.5)	6 (6.6)
One	119 (47.0)	49 (53.8)
Two or more	93 (36.8)	33 (36.3)
Unknown	12 (4.7)	3 (3.3)
Siblings with VI, n (%)		
Yes	41 (16.2)	21 (23.1)
No	171 (67.6)	61 (67.0)
N/A (no/unknown siblings)	41 (16.2)	9 (9.9)

### Item analyses

Table 2 presents the distribution of responses over the response categories for the Dutch sample and the UK sample. The response option ‘not applicable’ was treated as a missing value. As such, four items in the Dutch sample had missing scores > 20% (“using the computer for homework”, “taking part in science classes”, “taking part in geography classes”, and “watching plays and shows in the theatre”) and these items were removed. None of the items had floor or ceiling effects, and in all items all four response categories were endorsed. However, infrequent endorsement of the response option ‘very difficult or impossible’ in almost all items in the Dutch sample motivated the collapsing of response options ‘very difficult or impossible’ and ‘difficult’. There were no item pairs displaying high inter-item correlations (> 0.7). There were significant differences in the distribution of responses between the Dutch and the UK sample for all but five items. In general, the Dutch sample was more likely to opt for the response options 1 or 2 (‘very easy’ or ‘easy’) and less likely to opt for the response options 3 or 4 (‘difficult’ or ‘very difficult/impossible’) than the UK sample. Matching the Dutch sample on UK inclusion criteria (i.e. age 10–15 years and VI logMAR ≥0.48; *n* = 63 for Dutch sample and *n* = 85 for UK sample) did not influence these results.

**Table 2:** Differences in distribution of responses over the response categories for the Dutch sample (*n* = 253) and the UK sample (*n* = 91)

Item	Item content	Distribution of responding population Dutch sample (%) over the response options^a^				Missing responses Dutch sample (%)	Distribution of responding population UK sample (%) over the response options^a^				Missing responses UK sample (%)	*P*-value
		1	2	3	4		1	2	3	4		
FV_1	Watching TV	43.6	50.0	4.8	1.6	1.2	28.1	42.7	25.8	3.4	2.2	< 0.001
FV_2	Playing video and computer games	50.4	42.8	5.9	0.8	6.7	23.9	47.7	20.5	8.0	3.3	< 0.001
FV_3	Playing other games, e.g. board games or card games	35.4	53.6	10.5	0.4	6.3	21.3	34.8	38.2	5.6	2.2	< 0.001
FV_4	Using the computer for homework	35.8	56.2	7.3	0.7	45.8	26.1	52.3	17.0	4.5	3.3	0.020
FV_5	Reading food packets, labels or recipes	10.2	31.3	36.6	22.0	2.8	6.0	14.3	40.5	39.3	7.7	0.002
FV_6	Doing household chores, e.g. washing up	33.6	57.3	7.7	1.4	13.0	20.0	52.5	18.8	8.8	12.1	< 0.001
FV_7	Telling the time on a wrist watch	34.1	37.4	23.8	4.7	15.4	13.8	34.5	32.2	19.5	4.4	< 0.001
FV_8	Telling the time on a wall clock	26.8	45.2	17.6	10.5	5.5	11.2	28.1	30.3	30.3	2.2	< 0.001
FV_9	Using the computer for lessons	42.1	52.3	5.1	0.5	14.6	23.6	56.2	15.7	4.5	2.2	< 0.001
FV_10	Reading small print text books, worksheets and exam papers	11.6	28.9	35.5	24.0	4.3	3.4	13.6	38.6	44.3	3.3	< 0.001
FV_11	Reading enlarged text books, worksheets and exam papers	47.9	43.8	2.5	5.8	5.1	42.0	42.0	10.2	5.7	3.3	0.030
FV_12	Drawing or painting	32.5	56.3	8.3	2.9	5.1	30.3	33.7	31.5	4.5	2.2	< 0.001
FV_13	Reading hand writing	10.4	44.6	30.7	14.3	0.8	13.6	12.5	53.4	20.5	3.3	< 0.001
FV_14	Seeing the board in the class	25.0	46.0	19.4	9.7	2.0	8.2	23.5	35.3	32.9	6.6	< 0.001
FV_15	Recognizing people, e.g. in school corridors	32.4	48.6	15.0	4.0	0.0	15.9	33.0	33.0	18.2	3.3	< 0.001
FV_16	Recognizing other people’s facial expressions	23.4	45.6	20.6	10.3	0.4	21.8	28.7	28.7	20.7	4.4	0.008
FV_17	Finding friends in the playground	21.6	43.6	30.8	4.0	1.2	8.0	28.4	36.4	27.3	3.3	< 0.001
FV_18	Taking part in science classes	23.7	60.4	13.7	2.2	45.1	23.3	51.1	20.0	5.6	1.1	0.261
FV_19	Taking part in geography classes	24.1	56.0	16.3	3.6	34.4	18.2	44.2	28.6	9.1	15.4	0.027
FV_20	Taking part in math classes	27.2	49.6	21.2	2.0	1.2	22.2	52.2	21.1	4.4	1.1	0.528
FV_21	Taking part in PE	39.3	50.4	9.4	0.8	3.6	20.7	35.6	35.6	8.0	4.4	< 0.001
FV_22	Taking part in English/Dutch classes	30.5	55.0	12.4	2.0	1.6	20.2	57.3	20.2	2.2	2.2	0.150
FV_23	Keeping up with the teacher in lessons	21.8	53.2	22.6	2.4	0.4	21.1	37.8	35.6	5.6	1.1	0.023
FV_24	Keeping up with other students in class	23.9	52.6	22.3	1.2	0.8	23.1	33.0	39.6	4.4	0.0	0.001
FV_25	Getting around the school by yourself	43.3	50.4	6.0	0.4	0.4	42.9	41.8	13.2	2.2	0.0	0.047
FV_26	Getting around outdoors by yourself	36.5	52.0	10.7	0.8	0.4	17.2	41.4	34.5	6.9	4.4	< 0.001
FV_27	Reading signs and posters at stations or shops	19.2	40.8	30.8	9.2	5.1	13.6	26.1	30.7	29.5	3.3	< 0.001
FV_28	Getting around in crowds by yourself	12.1	32.0	44.1	11.7	2.4	11.4	19.0	39.2	30.4	13.2	0.001
FV_29	Seeing small moving objects, e.g. balls	14.6	40.3	30.4	14.6	0.0	10.3	11.5	40.2	37.9	4.4	< 0.001
FV_30	Seeing large moving objects, e.g. cars passing	39.1	45.1	10.7	5.1	0.0	30.7	50.0	12.5	6.8	3.3	0.546
FV_31	Using the escalators	39.6	49.4	9.4	1.6	3.2	40.4	38.2	18.0	3.4	2.2	0.077
FV_32	Playing team sports, e.g. football, without adaptations	27.6	50.7	18.7	3.0	19.8	18.4	21.8	33.3	26.4	4.4	< 0.001
FV_33	Watching films in the cinema	40.5	51.4	7.3	0.9	13.0	34.8	40.4	19.1	5.6	2.2	0.001
FV_34	Watching plays and shows in the theatre	26.2	55.9	14.9	3.0	20.2	15.0	38.8	31.3	15.0	12.1	< 0.001
FV_35	Reading price tags	16.5	49.6	24.2	9.7	2.0	12.0	30.1	33.7	24.1	8.8	< 0.001
FV_36	Finding correct money to pay	22.6	55.7	20.0	1.7	9.1	22.4	40.0	25.9	11.8	6.6	< 0.001

^a^1: very easy; 2: easy; 3: difficult; 4: very difficult/impossible

### Calibration of the FVQ_CYP_NL

The acceleration factor suggested a one-factor solution for the Dutch data. Principal components of the one-factor solution were all positive and moderate to large. Inspection of item and factor content gave no reason for multidimensional solutions. The first factor accounted for 33% of the variance, whereas the second factor accounted for 5% of the variance; thus, the ratio of explained variance by the first and second factor is 6.6, which is higher than the required minimum of 4 [45]. It was therefore concluded that the 32 items stem from a unidimensional scale. Examination of the residual correlation matrix showed that one out of 496 item pairs (0.2%) showed excess item covariation (> 0.25), violating the assumption of local independence (“keeping up with the teacher in lessons” – “keeping up with other students in class”). However, since the violation was not very severe (0.267), it was decided not to remove one of the items. Monotonicity analysis (piecewise assessment in sets of 16 items in order to retain samples after list wise deletion) showed that all items complied with monotonicity, and none of the items had a Loevinger H coefficient below 0.3, indicating sufficient scalability.

Five items were removed after the first application of the GRM: “reading food packets, labels or recipes”, “doing household chores, e.g. washing up”, “telling the time on a wrist watch”, “drawing or painting”, and “keeping up with the teacher in lessons”. These items were removed because they provided very little information (i.e. little precision/discrimination) and/or because they covered the same area on the disability trait as another item, but provided less information and/or provided information over a smaller range of the disability trait. Content validity, item relevance and similarities with other items were also considered. Three additional items were removed after the second fit of the GRM (“taking part in math classes”, “taking part in physical education”, and “taking part in Dutch language classes”), mainly because they still provided very little information.

**Fig. 1:**
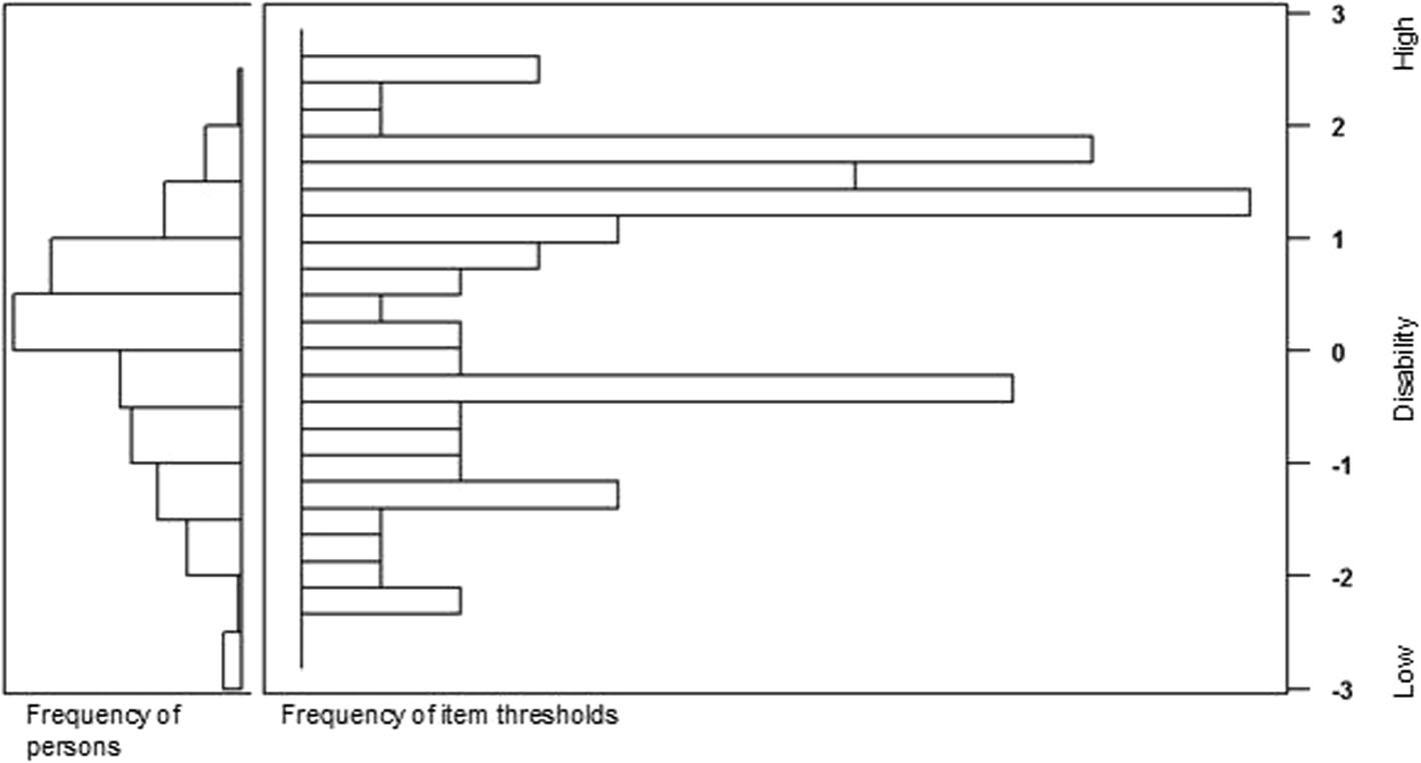
Item-person map of the 24 item FVQ_CYP_NL

The Likelihood Ratio test showed that the full GRM outperformed the polytomous Rasch model for the 24 items (LRT = 40.0, *p* = 0.015). The fit indices reflected adequate overall model fit of the 24 items to the GRM: RMSEA = 0.061, SRMR = 0.062, TLI =0.965, and CFI = 0.968. Table 3 summarizes GRM item parameters, information and fit statistics of the FVQ_CYP_NL. Item discrimination ranged from 1.11 to 2.27. The item with the lowest discrimination was “reading small print text books, worksheets and exam papers”, and the item with the highest discrimination was “reading enlarged text books, worksheets and exam papers”. Item threshold parameters ranged from − 2.26 to 2.60. Item information ranged from 1.76 to 4.26, and total information of the 24 items was 65.32. All items fitted the GRM at the *p* < 0.01 level. Despite the fact that some items still provided little information, further item removal was considered unfavourable given the location of these items on the disability trait and for reasons of content validity. The item-person map shows that items are distributed almost entirely across the disability trait. The thetas of respondents adequately match the item thresholds, although there are no items for persons with low levels of disability (Fig. 1).

**Table 3:** GRM item characteristics for the 24 item FVQ_CYP_NL (*n* = 253)

Item	Item content	Discrimination α	Threshold β1	Threshold β2	Item information	X^2^	*P*-value
FV_1	Watching TV	1.32	−0.26	2.49	2.45	8.17	0.52
FV_2	Playing video and computer games	1.19	− 0.02	2.56	2.12	13.04	0.22
FV_3	Playing other games, e.g. board games or card games	1.41	−0.57	1.89	2.58	12.41	0.26
FV_8	Telling the time on a wall clock	1.74	−0.84	0.85	3.06	13.45	0.20
FV_9	Using the computer for lessons	1.29	−0.36	2.60	2.41	11.51	0.18
FV_10	Reading small print text books, worksheets and exam papers	1.11	−2.26	−0.43	1.76	11.69	0.39
FV_11	Reading enlarged text books, worksheets and exam papers	2.27	−0.004	1.70	4.26	8.95	0.18
FV_13	Reading hand writing	1.22	−2.24	0.24	2.17	9.03	0.53
FV_14	Seeing the board in the class	1.42	−1.05	0.88	2.46	19.18	0.12
FV_15	Recognizing people, e.g. in school corridors	1.50	−0.67	1.33	2.66	11.86	0.46
FV_16	Recognizing other people’s facial expressions	1.67	−1.03	0.74	2.96	19.43	0.05
FV_17	Finding friends in the playground	1.36	−1.24	0.67	2.33	15.44	0.22
FV_24	Keeping up with other students in class	1.28	−1.19	1.20	2.27	14.36	0.35
FV_25	Getting around the school by yourself	1.72	−0.23	2.11	3.24	14.04	0.05
FV_26	Getting around outdoors by yourself	1.80	−0.44	1.63	3.34	9.00	0.44
FV_27	Reading signs and posters at stations or shops	1.64	−1.28	0.40	2.84	13.22	0.21
FV_28	Getting around in crowds by yourself	1.25	−2.05	−0.23	2.05	15.07	0.18
FV_29	Seeing small moving objects, e.g. balls	1.26	−1.80	0.23	2.15	14.90	0.19
FV_30	Seeing large moving objects, e.g. cars passing	1.82	−0.34	1.36	3.25	14.89	0.19
FV_31	Using the escalators	1.58	−0.34	1.80	2.89	13.38	0.15
FV_32	Playing team sports, e.g. football, without adaptations	1.58	−0.86	1.16	2.85	12.22	0.27
FV_33	Watching films in the cinema	1.90	−0.37	1.77	3.59	8.50	0.29
FV_35	Reading price tags	1.61	−1.45	0.64	2.93	15.53	0.11
FV_36	Finding correct money to pay	1.48	−1.16	1.17	2.70	9.81	0.46

### Differential item functioning and known-group validity

**Fig. 2:**
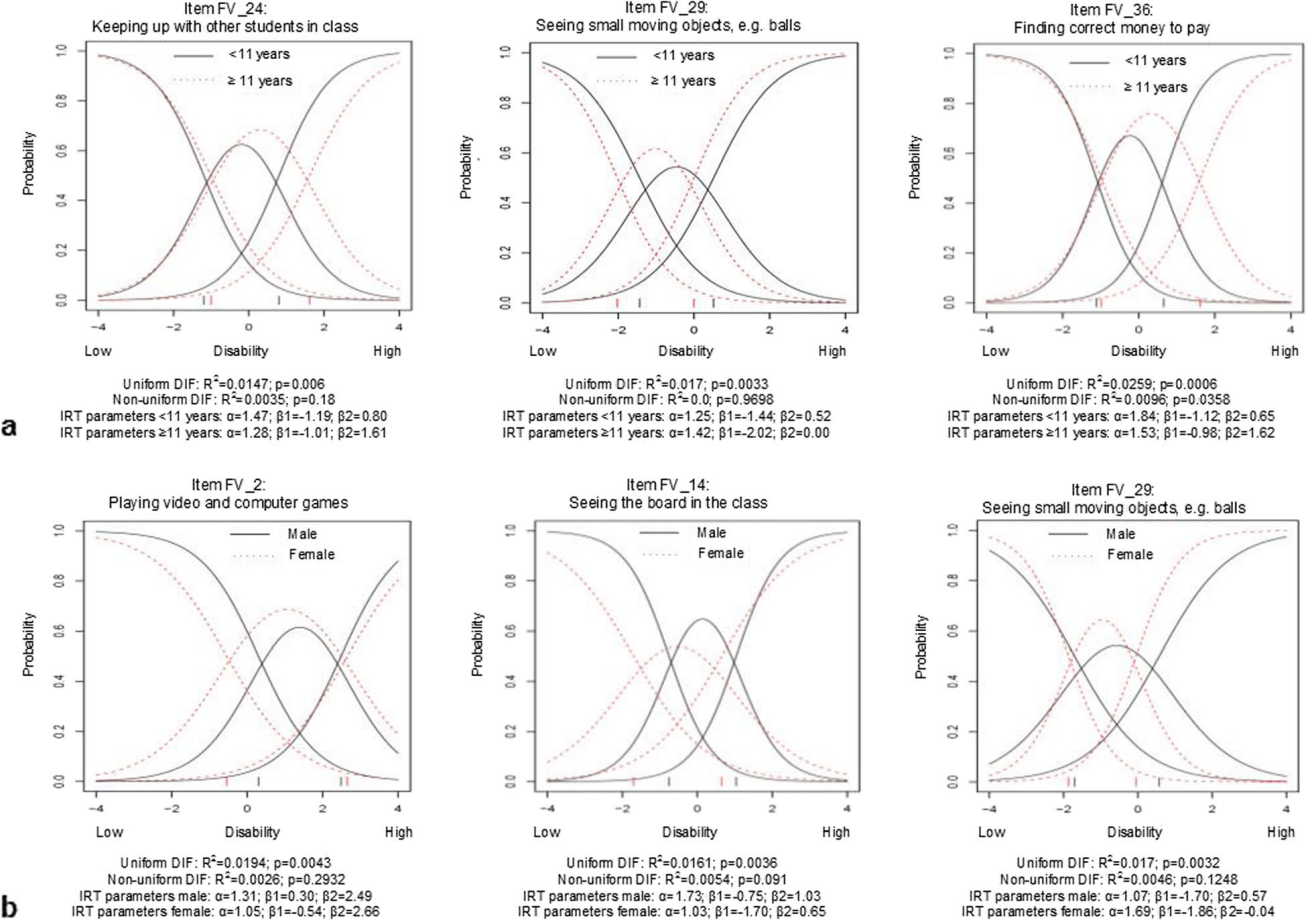
Item response functions, McFadden’s pseudo R^2^ and *p*-values, and IRT parameters for items displaying DIF for age (**a**) and gender (**b**) (*n* = 253)

After two iterations, analysis of DIF for age indicated three items with some level of DIF, which was all uniform (Fig. 2a). However, change in McFadden’s R^2^ was below 2% for two of the three items. For two items (“keeping up with other students in class” (R^2^ = 0.0147) and “finding correct money to pay” (R^2^ = 0.0259)), younger children were more likely to endorse higher response categories (signifying higher levels of difficulty) compared to older children. Item response functions suggest that uniform DIF was due to second category threshold values being smaller for the younger group than for the older group for both items. For one item (“seeing small moving objects, e.g. balls” (R^2^ = 0.017)), older children were more likely to endorse higher response categories. Here, the item response functions suggest that the category threshold values were both smaller for the older group than for the younger group. Analysis of DIF for gender also indicated three items with some level of DIF after three iterations (Fig. 2b), but change in McFadden’s R^2^ was below 2% (“playing video and computer games” (R^2^ = 0.0194), “seeing the board in the class” (R^2^ = 0.0161), and “seeing small moving objects, e.g. balls” (R^2^ = 0.017)). According to χ^2^ tests, all items displayed uniform DIF. However, item response functions revealed non-uniform DIF, indicated by differences in slope parameters.

**Fig. 3:**
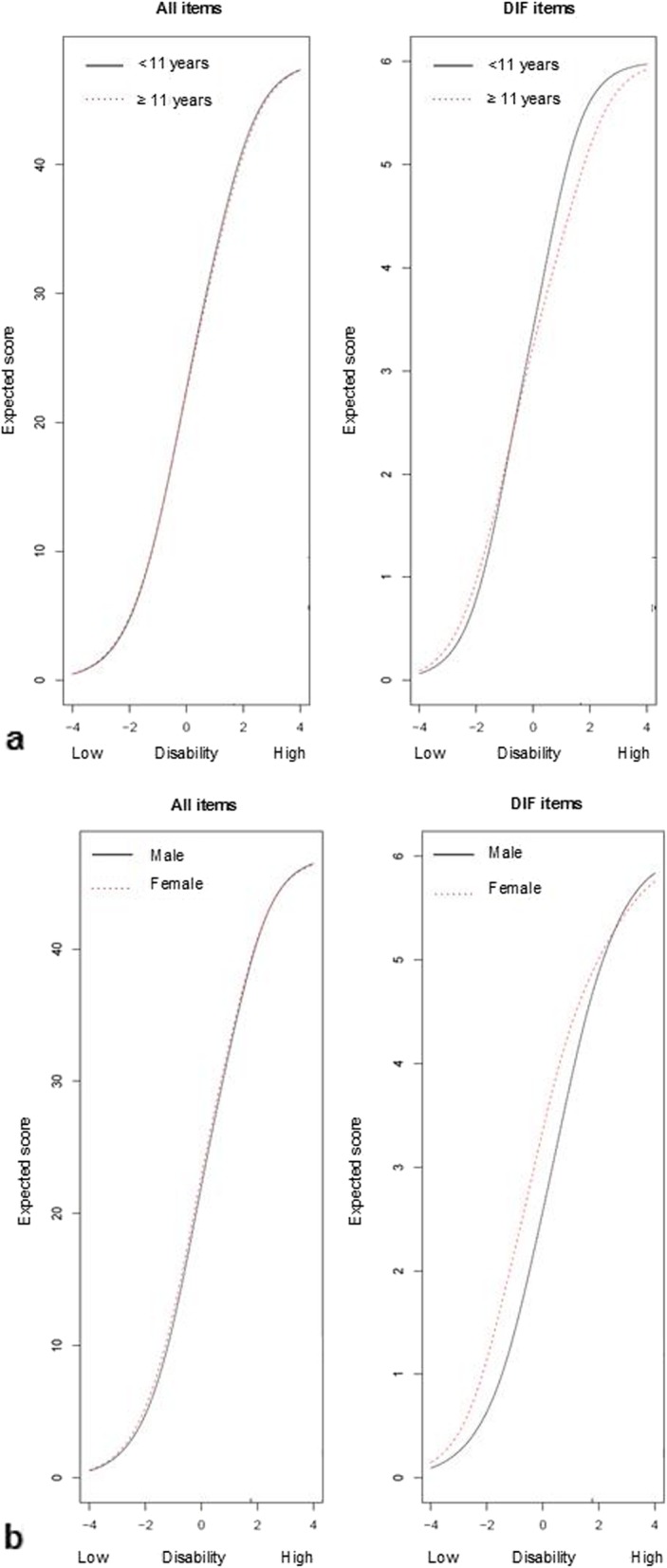
Total impact of DIF on the test characteristic curve (TCC) for age (**a**) and gender (**b**) (*n* = 253)

Figure 3a shows the total impact of DIF for age on the test characteristic curve (TCC), and Fig. 3b the total impact of DIF for gender. The TCC shows the relation between the total scores (y-axis) and thetas (x-axis). The left graphs show the impact on the test score for all items, whereas the right graphs show the impact of only those items with DIF. The curves show that the total score is the same for both age groups and genders, indicating minimal impact of DIF by age and gender.

Known-group validity was established for groups that differ on level of VI and gender. Those with severe VI/blindness had significantly higher thetas than those with moderate VI and mild VI (p= 0.002 and *p*<0.001 respectively), indicating that they experienced more disability and the FVQ_CYP_NL was able to discriminate them. Females had significantly higher thetas than males (*p*=0.008), and no significant differences were found in thetas between those with and without other impairments.

## Discussion

This study reports the cross-cultural adaptation of the original UK version of the FVQ_CYP into Dutch and its important psychometric properties. The FVQ_CYP is a PROM which measures functional vision of children and adolescents with VI [18]. Following standardized translation processes, the original English instrument translated well into Dutch resulting in a new Dutch version of the questionnaire – the FVQ_CYP_NL. However, the cross-cultural validation in the Dutch population using the measurement model specifications and assumptions used in the original UK study was not straightforward. Since some adaptations needed to be made to achieve model fit of the Dutch version, it can be argued whether the FVQ_CYP_NL still measures the same construct as the original UK FVQ_CYP. However, both versions proved to be unidimensional scales with a broad coverage of items measuring children’s self-assessed ability to endorse vision-dependent tasks. The FVQ_CYP_NL has high measurement precision, is targeted adequately to the abilities of children and adolescents aged 7–17 years with different levels of VI, and can discriminate between these levels.

We originally planned to perform ‘strict’ cross-cultural validation of the FVQ_CY_NL by applying the same criteria for item analyses as used in the validation study of the UK questionnaire [18], using the RSM, and conducting DIF analysis for country. This would have allowed direct cross-cultural comparisons in future studies as well as pooling the data from the two countries for instance in the context of trials of new therapies or interventions.

Interestingly, we found a number of differences in the psychometric performance of the instrument versions of the two countries. There were differences in the distribution of missing responses and response patterns between the Dutch data and the UK data. Some items had high missing responses in the Netherlands, but not in the UK, and Dutch children were less likely to opt the response category ‘very difficult/impossible’. There are a number of possible reasons for these differences. Firstly, the difference in instrument performance between countries may have been driven by differences in the population due to the broader age range and less restrictions in degree of vision impairment in the Dutch sample. There were also differences in the presence of comorbidity between the samples. Matching the samples did not improve the results. Secondly, differences might have been influenced by different modes of administration. Data in the original UK study had been collected as self-report and self-completion with questionnaires returned by post [18], whereas in the Netherlands data was collected using face-to-face interviews via home visits. Face-to-face interviews are known to be more prone to social desirability bias and yes-saying bias, while respondents are less willing to disclose sensitive information [46]. Thirdly, the FVQ_CYP UK version was developed within a specific population which drove the questionnaire content, including semi-structured and cognitive interviews with children and adolescents to develop and shape the instrument items and formats. Thus, the FVQ_CYP may more accurately capture the UK children’s functional vision because the content is more relevant to them both with respect to age and level of acuity: interviewing Dutch children to develop a similar instrument de novo may have resulted in a different set of items. Despite the mismatch between the Dutch population and the intended target population of the FVQ_CYP with respect to age and level of vision impairment, we decided to use the FVQ_CYP because it currently is the most robust instrument to measure functional vision in children.

Besides the differences in psychometric performance, there was non-invariance at the model level; the RSM did not fit the Dutch data, whereas fit for the UK data was satisfactory. The RSM assumes that the discrimination parameter (i.e. the slope) is equal across all items (and therefore this model belongs to the Rasch family), and that the thresholds for each category response are also equal across items [40]. These assumptions make the RSM among the more restrictive IRT models. However, the RSM can tolerate smaller sample sizes than the commonly used GRM, which has fewer assumptions and is more flexible [40]. The sample sizes of both the UK data and the Dutch data were modest and therefore rather limited for the advanced analyses conducted in this study [24, 47]; furthermore, the difference in sample size between the Dutch data and UK data might have contributed to the non-invariance at the model level, as any model fit is contingent upon sample size. Besides unsatisfactory overall fit of the RSM to the Dutch data, various tests for item fit indicated misfit of items to the model. After iteratively removing those items with most misfit, the goodness-of-fit test was suggestive for satisfactory model fit of the RSM. However, by then only 12 items were maintained in the FVQ_CYP_NL, which was considered undesirable because of the threat to face and content validity. Matching of the Dutch sample to UK inclusion criteria led to more violations to IRT assumptions. Model fit improved, but 11 items were excluded in the analysis because of inappropriate response patterns, and tests for item fit still indicated items with misfit.

Measurement invariance implies that the association between test scores and latent traits of persons is unconditional on group affiliation or time of measurement [48]. The non-invariance at the model level already implied that there would be DIF for country or non-invariance at item level as well. The assumption of measurement invariance rarely holds, especially when parameters are expected to be exactly the same across groups. But even when applying less strict criteria, the occurrence of measurement invariance is often ignored, and populations are compared even though there is no psychometric basis for it, thereby introducing potential bias [48]. Most studies only report the results of DIF analyses in assessing cross-cultural validity, not taking into account the measurement model specifications and assumptions used in the original validation study. We chose not to ignore measurement invariance, and used a different IRT model to investigate the FVQ_CYP_NL and compare it to the original FVQ_CYP. As such, the two instruments were not calibrated on the same scale and, consequently, item parameters of the original UK instrument and the FVQ_CYP_NL are not comparable. In addition, changes were made in the number of items of the FVQ_CYP_NL, and in response options. Therefore, someone with the same true theta on the disability trait who completes the FVQ_CYP_NL will have a different score when completing the original FVQ_CYP.

Application of the GRM resulted in the identification of eight items that contributed very little information or covered the same area on the disability trait as another item while providing less information, and therefore these were removed. Together with the four items that had too many missing responses, this resulted in an instrument containing 24 items. Some of the items which were deleted might have been less relevant for younger children (i.e. “using the computer for homework”, “doing household chores, e.g. washing up”, and “reading food packets, labels or recipes”). The item “telling the time on a wrist watch” might have been superseded by modern technology, e.g. the use of smartphones. This might even be true for younger children, as 68% of the 10-year olds in the Netherlands had a smartphone in 2017 [49]. The large variability of mandatory classes in the Dutch school system might have caused that items on individual classes were less relevant to Dutch children, contributing to the high number of missing values. For instance, science classes en geography are only mandatory for older children at some point, but the age at which these classes are mandatory is also dependent on school level. The item “drawing or painting” might have been less relevant for older children, as art is an elective class at several educational levels and ages in the Dutch school system. Furthermore, it was hypothesized that the influence of impaired vision on school performance is probably better captured by items such as reading text books, seeing the board and keeping up with other students, than by items about individual classes, for which endorsement might be more driven by other factors, such as individual talents or pedagogical methods. This hypothesis supported the removal of the items “taking part in math classes”, “taking part in physical education”, and “taking part in Dutch language classes” and the other class-specific items mentioned above.

The item thresholds of the final instrument reflected a good coverage across the disability trait. The FVQ_CYP_NL seemed better targeted to children and adolescents with higher thetas at the disability trait, and there might be a need for more difficult items. However, this was also expected, because the FVQ_CYP was originally developed with and for children with more severe visual impairment than the Dutch sample. This study shows that, with appropriate modification, it is also possible to administer the questionnaire to children and adolescents outside the original 10–15 years age interval, and to children and adolescents with less severe visual impairment. This was already anticipated by the authors of the original FVQ_CYP, who are currently completing development and an additional assessment of psychometric properties of age-appropriate versions applicable to a wider age range [18].

The DIF analyses identified three items with uniform DIF for age and three items (one overlapping) with non-uniform DIF for gender (although results of χ^2^ tests point towards uniform DIF). However, DIF had minimal impact on the total score, and therefore we concluded that item differences for age groups and gender are negligible, and removing these items was not necessary. Although one could argue that the impact of DIF on the total test score is most important, we believe it is also important to mention DIF per item. When for example selecting items for a short form or computer adaptive test (CAT), it is important to know which items display DIF, and omitting these items in the short form or CAT would be preferred.

Infrequent endorsement of the fourth response category motivated collapsing the third and fourth category. However, adding the ‘not applicable’ response option might have caused attrition in the data, because children might have opted for the ‘not applicable’ category where they also could have opted for ‘very difficult/impossible’. This was also speculated to be the case in the validation of the original instrument in the UK, and with frequent endorsement of the ‘not applicable’ option resulting in a high proportion of missing data, the authors subsequently decided to remove ‘not applicable’ as a response option from the instrument [18]. However, because of the wider age range in the Dutch sample, the response option ‘not applicable’ was warranted in the FVQ_CYP_NL, as children aged for example 7 years do not have certain classes in school or receive homework. The deletion of items ensured that the items in the final 24 item version of the FVQ_CYP_NL are probably applicable to the entire age range (e.g. 7–17 years), and therefore the response option ‘not applicable’ could be deleted again, in order to prevent entanglement with the fourth response category. Consequently, it should be tested whether the fourth response category can exist independently, or whether collapsing it with the third category is still necessary. Furthermore, 10 participants commented that the distance between the second and third category (i.e. ‘easy’ and ‘difficult’) was too large, and an answer option in between is necessary. In a large share of the participants, the interviewers also noted that children were often in doubt on whether to opt for ‘easy’ or ‘difficult’ in at least some items, and therefore addition of the response option ‘little difficult’ would be desirable.

## Conclusions

In conclusion, non-invariance at the model level, small sample sizes, and differences in population characteristics and distribution of responses posed challenges to the standard cross-cultural validation process. However, although this imposes limitations to direct comparability of the FVQ-CYP between the Netherlands and UK, by using a GRM, we have established validity of the FVQ_CYP_NL as a stand-alone instrument for use in the Netherlands (thus the FVQ_CYP UK version served as the building block). The Dutch adapted FVQ_CYP – the FVQ_CYP_NL – is a unidimensional scale with high measurement precision and broad coverage of items measuring children’s functional vision. Deletion of items ensured that only those items most applicable to the Dutch setting and providing high information were included in the final questionnaire. This study provides detailed information on item parameters, and shows that the FVQ_CYP_NL is targeted adequately to the abilities of children and adolescents aged 7–17 with different levels of VI. In its current form the FVQ_CYP_NL is a short, easy to administer instrument, with sound psychometric properties, which can be used to assess the self-reported level of difficulty in performing vision-dependent activities in children and adolescents with visual impairment. However, further evaluation of psychometric properties such as the application and functioning of the recommended response categories, construct validity, test-retest reliability, and responsiveness is necessary.

Our study has implications for cross-cultural use of instruments in general. Given the scarcity of measures for children and adolescents in ophthalmology and the challenges in developing instruments de novo with heterogeneous and numerically small clinical populations, there is a value in using well developed instruments and adapting them cross-culturally. However, care must be taken that rigorous, standard cross-cultural validation processes are followed. Even when instruments are invariant at model or item level, it is possible to have language versions of an instrument that are reliable and valid for use in each country but differ extensively in wording or are even comprised of different items from item banks, that demonstrate identical response functions, facilitating cross-cultural use [50]. Our findings highlight that instruments cannot simply be taken and translated into another language whilst expecting that the underlying psychometric assumptions will remain across different cultures. We argue that when performing cross-cultural validations, researchers should assess invariance at both model level and item level (i.e. DIF analyses), and confirm that language versions function similarly in different countries. If this is not the case, considering the original instrument as the building blocks and assessing psychometric properties of the new language version using more liberal assumptions is recommended.

## Data Availability

The datasets used and/or analysed during the current study are available from the corresponding author on reasonable request.

## Abbreviations

CFI: Comparative fit index
CRC: Category response curve
DIF: Differential item functioning
FVQ_CYP: Functional Vision Questionnaire for Children and Young People
FVQ_CYP_NL: Dutch version of the Functional Vision Questionnaire for Children and Young People
GRM: Graded response model
IIC: Item information curve
IRT: Item response theory
PAI-CY: Participation and Activity Inventory for Children and Youth
PCA: Principal component analysis
PROM: Patient-reported outcome measure
RMSEA: Root mean square error of approximation
RSM: Rating scale model
SRMR: Standardized root mean square residual
TCC: Test characteristic curve
TLI: Tucker-Lewis index
UK: United Kingdom
VI: Visual impairment
WHO: World Health Organisation

## References

[1] Black N, Jenkinson C (2009). Measuring patients’ experiences and outcomes. BMJ.

[2] Smith SC, Cano S, Lamping DL, Staniszewska S, Browne J, Lewsey J (2005). Patient-reported outcome measures (PROMs) for routine use in treatment Centres: recommendations based on a review of the scientific evidence. Final report to the Department of Health.

[3] Black N (2013). Patient reported outcome measures could help transform healthcare. BMJ.

[4] Breitscheidel L, Stamenitis S (2009). Using patient-reported outcome assessments in clinical practice and their importance in risk management. J Med Econ.

[5] Greenhalgh J (2009). The applications of PROs in clinical practice: what are they, do they work, and why?. Qual Life Res.

[6] Frost NA, Sparrow JM, Durant JS, Donovan JL, Peters TJ, Brookes ST (1998). Development of a questionnaire for measurement of vision-related quality of life. Ophthalmic Epidemiol.

[7] Hassell JB, Weih LM, Keeffe JE (2000). A measure of handicap for low vision rehabilitation: the impact of vision impairment profile. Clin Exp Ophthalmol.

[8] Mangione CM, Lee PP, Gutierrez PR, Spritzer K, Berry S, Hays RD (2001). Development of the 25-item National eye Institute visual function questionnaire. Arch Ophthalmol.

[9] Horowitz A, Reinhardt JP (1998). Development of the adaptation to age-related vision loss scale. J Visual Impair Blin.

[10] Wolffsohn JS, Cochrane AL (2000). Design of the low vision quality-of-life questionnaire (LVQOL) and measuring the outcome of low-vision rehabilitation. Am J Ophthalmol.

[11] Steinberg EP, Tielsch JM, Schein OD, Javitt JC, Sharkey P, Cassard SD (1994). The Vf-14 - an Index of Functional Impairment in Patients with Cataract. Arch Ophthalmol-Chic.

[12] Lundstrom M, Roos P, Jensen S, Fregell G (1997). Catquest questionnaire for use in cataract surgery care: Description, validity, and reliability. J Cataract Refr Surg.

[13] Gothwal VK, Lovie-Kitchin JE, Nutheti R (2003). The development of the LV Prasad-functional vision questionnaire: a measure of functional vision performance of visually impaired children. Invest Ophthalmol Vis Sci.

[14] Gothwal VK, Sumalini R, Bharani S, Reddy SP, Bagga DK (2012). The second version of the L. V. Prasad-functional vision questionnaire. Optom Vis Sci.

[15] Huang Jinhai, Khadka Jyoti, Gao Rongrong, Zhang Sifang, Dong Wenpeng, Bao Fangjun, Chen Haisi, Wang Qinmei, Chen Hao, Pesudovs Konrad (2016). Validation of an instrument to assess visual ability in children with visual impairment in China. British Journal of Ophthalmology.

[16] Özen Tunay Zuhal, Çalişkan Deniz, Öztuna Derya, İdil Aysun (2015). Validation and reliability of the Cardiff Visual Ability Questionnaire for Children using Rasch analysis in a Turkish population. British Journal of Ophthalmology.

[17] Khadka J, Ryan B, Margrain TH, Court H, Woodhouse JM (2010). Development of the 25-item Cardiff visual ability questionnaire for children (CVAQC). Br J Ophthalmol.

[18] Tadic V, Cooper A, Cumberland P, Lewando-Hundt G, Rahi JS (2013). Vision-related quality of life G. development of the functional vision questionnaire for children and young people with visual impairment: the FVQ_CYP. Ophthalmology.

[19] Elsman EBM, van Nispen RMA, van Rens GHMB (2017). Feasibility of the Participation and Activity Inventory for Children and Youth (PAI-CY) and Young Adults (PAI-YA) with a visual impairment: a pilot study. Health Qual Life Out.

[20] Beaton DE, Bombardier C, Guillemin F, Ferraz MB (2000). Guidelines for the process of cross-cultural adaptation of self-report measures. Spine.

[21] WHO (2010). International Statistical Classification of Diseases and Related Health Problems 10th Revision. Version 2010. Chapter VII, H54: visual impairment including blindness.

[22] R: A language and environment for statistical computing. Vienna. R Core Team. R Foundation for Statistical Computing; 2017. https://www.R-project.org/:

[23] IBM Corp N (2013). IBM SPSS Statistics for Windows, Version 22.0.

[24] Edelen MO, Reeve BB (2007). Applying item response theory (IRT) modeling to questionnaire development, evaluation, and refinement. Qual Life Res.

[25] Reeve BB, Hays RD, Chang CH, Perfetto EM (2007). Applying item response theory to enhance health outcomes assessment. Qual Life Res.

[26] Raîche Gilles, Walls Theodore A., Magis David, Riopel Martin, Blais Jean-Guy (2013). Non-Graphical Solutions for Cattell’s Scree Test. Methodology.

[27] Pilkonis PA, Choi SW, Reise SP, Stover AM, Riley WT, Cella D (2011). Item banks for measuring emotional distress from the patient-reported outcomes measurement information system (PROMIS(R)): depression, anxiety, and anger. Assessment.

[28] Loevinger J (1948). The technic of homogeneous tests compared with some aspects of scale analysis and factor analysis. Psychol Bull.

[29] Meijer RR, Baneke JJ (2004). Analyzing psychopathology items: a case for nonparametric item response theory modeling. Psychol Methods.

[30] Sijtsma K, Meijer RR, van der Ark LA (2011). Mokken scale analysis as time goes by an update for scaling practitioners. Pers Indiv Differ.

[31] Mair P, Hatzinger R (2007). Extended Rasch modeling: the eRm package for the application of IRT models in R. J Stat Softw.

[32] Samejima F (1969). Estimation of Latent Ability Using a Response Pattern of Graded Scores. Psychometrika.

[33] Samejima F, Van der Linden W, Hambleton RK (1997). Graded response model. Handbook of modern item response theory.

[34] Rizopoulos D (2006). ltm: An R package for latent variable modeling and item response theory analyses. J Stat Softw.

[35] Bond TG, Fox CM (2007). Applying the Rasch model - fundamental measurement in the human sciences (2ed.).

[36] Chalmers RP (2012). Mirt: a multidimensional item response theory package for the R environment. J Stat Softw.

[37] Hu LT, Bentler PM (1999). Cutoff criteria for fit indexes in covariance structure analysis: conventional criteria versus new alternatives. Struct Equ Modeling.

[38] Bock RD (1972). Estimating item parameters and latent ability when responses are scored in two or more nominal categories. Psychometrika.

[39] Yen WM (1981). Using simulation results to choose a latent trait model. Appl Psychol Meas.

[40] Nguyen TH, Han HR, Kim MT, Chan KS (2014). An introduction to item response theory for patient-reported outcome measurement. Patient..

[41] Irribarra TD, Freund R (2014). Wright Map: IRT item-person map with ConQuest integration.

[42] De Vet HCW, Terwee CB, Mokkink LB, Knol DL (2011). Measurement in Medicine: a practical guide.

[43] Choi SW, Gibbons LE, Crane PK (2011). Lordif: an R package for detecting differential item functioning using iterative hybrid ordinal logistic regression/item response theory and Monte Carlo simulations. J Stat Softw.

[44] Choi SW, Gibbons LE, Crane PK (2011). Lordif: an R package fo rDetecting differential item functioning using iterative hybrid ordinal logistic regression/item response theory and Monte Carlo simulations. J Stat Softw.

[45] Reeve BB, Hays RD, Bjorner JB, Cook KF, Crane PK, Teresi JA (2007). Psychometric evaluation and calibration of health-related quality of life item banks: plans for the patient-reported outcomes measurement information system (PROMIS). Med Care.

[46] Bowling A (2005). Mode of questionnaire administration can have serious effects on data quality. J Public Health (Oxf).

[47] Tsutakawa RK, Johnson JC (1990). The Effect of Uncertainty of Item Parameter-Estimation on Ability Estimates. Psychometrika.

[48] Van De Schoot R, Schmidt P, De Beuckelaer A, Lek K, Zondervan-Zwijnenburg M (2015). Measurement invariance. Front Psychol.

[49] Kennisnet (2017). Monitor Youth and Media 2017 [Monitor Jeugd en Media 2017].

[50] Kucukdeveci AA, Sahin H, Ataman S, Griffiths B, Tennant A (2004). Issues in cross-cultural validity: example from the adaptation, reliability, and validity testing of a Turkish version of the Stanford health assessment questionnaire. Arthrit Rheum-Arthr.

